# Comparison of botulinum toxin with surgery for the treatment of acute acquired comitant esotropia and its clinical characteristics

**DOI:** 10.1038/s41598-019-50383-x

**Published:** 2019-09-25

**Authors:** Li-juan Lang, Yu Zhu, Zhi-gang Li, Guang-ying Zheng, Hai-ying Peng, Jun-bo Rong, Li-min Xu

**Affiliations:** 1 Department of ophtalmology, The First Affiliated Hospital of Zhengzhou University, Zhengzhou, 450052 China; 2 Henan Province People’s Hospital, Henan Provincial Ophthalmology Hospital, Zhengzhou, 450003 China

**Keywords:** Diseases, Medical research

## Abstract

We compared the therapeutic effects between botulinum toxin and surgery for acute acquired comitant esotropia (AACE) and analyze its clinical characteristics. The data of the 29 cases, who received treatment for AACE in the Ophthalmic Center of the First Affiliated Hospital of Zhengzhou University and Henan Provincial Ophthalmology Hospital between January 2016 and January 2017, were collected. The 29 cases with AACE were followed for 6 months or more, and received either botulinum toxin injection (group A with 13 cases) or squint correction (group B with 16 cases). The distant and near deviation angles were compared between the two groups before and after treatment. The success rate (total horizontal deviation of 10 prism diopters or less) and stereopsis were compared between the two groups at post-treatment 6 months. At the same time, the relations between distant and near deviation angles were analyzed among different myopia levels and different AACE types. Results indicated that he success rate was not significantly different at post-treatment 6 months (84.6% *vs* 81.3%, *P* = 1.00). The distant and near deviation angles were all significantly different one day and one month after treatment (all *P* < 0.05); but at post-treatment 6 months, they were not significantly different (all *P* > 0.05) between the two groups. There were no significant differences in the distant and near stereoacuity between the two groups at post-treatment 6 months (all *P* > 0.05). Among the 25 cases with myopia, the pre-treatment distant deviation angle was significantly higher than pre-treatment near deviation angle in the cases with myopia level >−2.5 D (*P* < 0.05), and the pre-treatment distant and near deviation angles were all significantly higher in the cases with type-IIAACE than in the cases with type-IIIAACE (all *P* < 0.05). This study suggests that Botulinum toxin is as effective as surgery in the treatment of AACE at post-treatment 6 months. For the cases with myopia level >−2.5 D, the pre-treatment distant deviation angle is significantly higher than pre-treatment near deviation angle; and both pre-treatment distant and near deviation angles are greater in the cases with type-IIAACE than in the cases with type-IIIAACE.

## Introduction

Acute acquired comitant esotropia (AACE), a subtype of esotropia, is acquired and rare. AACE mainly occurs in older children, adults and even the elderly. The clinical characteristics of AACE were (1) sudden-onset esotropia with diplopia which most cases can endure; (2) the diplopia with an equal picture interval in each direction; (3) recessive, intermittent or constant esotropia; (4) normal eye movements and equal squint deviation in each direction; and (5) a certain binocular visual function^1^.

For the treatment of AACE, there are conservative treatment, surgery^2–5^ and botulinum toxin injection^6^. For early AACE, the conservative treatment is usually adopted such as administration of neurotrophin, vasodilator and the vitamins, but the therapeutic effects of the conservative treatment are not marked. Therefore, surgical treatment or botulinum toxin injection is often used in clinics currently. However, whether there is a difference between the two therapies and which therapy is more superior for treatment of AACE? The aim of this study was to analyze clinical data of 29 cases with AACE, providing a basis for the treatment of AACE.

## Results

### General data

In this study, there were 13 cases in the group A and 16 cases in the group B. In the 13 cases of group A, 11 were male and 2 female, with a mean age of 12.61 ± 6.74 years (range 3–24). In the 16 cases of group B, 11 were male and 5 female, with a mean age of 20.18 ± 8.19 years (range 3–32). In the group A, the spherical equivalent was −1.73 ± 2.63 D in the right eye and −1.36 ± 2.62 D in the left eye. In the group B, the spherical equivalent was −3.11 ± 2.20 D in the right eye and −2.73 ± 1.96 D in the left eye. The pre-treatment distant deviation angle was 47.30 ± 22.69 PD in the group A and 39.66 ± 20.13 PD in the group B. The pre-treatment near deviation angle was 41.92 ± 27.65 PD in the group A and 32.66 ± 24.11 PD in the group B. There were no significant differences in all the general data between the two groups (all *P* < 0.05, Table 1).

**Table 1:** Comparison of pre-treatment general data between the group A and group B.

	Group A (n = 13)	Group B (n = 16)	*P* values
Age (year)	12.61 ± 6.74 (3~24)	20.18 ± 8.19 (3~32)	0.786
Male/female	11/2	11/5	0.156
Right spherical equivalent (D)	−1.73 ± 2.63	−3.11 ± 2.20	0.142
Left spherical equivalent (D)	−1.36 ± 2.62	−2.73 ± 1.96	0.266
Distant deviation angle (PD)	47.30 ± 22.69	39.66 ± 20.13	0.263
Near deviation angle (PD)	41.92 ± 27.65	32.66 ± 24.11	0.514

Note: PD: prism diopter.

### Comparison of distant and near deviation angles among different myopia levels

Based on different myopia levels, the 25 cases with myopia were divided into 3 groups including myopia ≤−2.5 D group, −2.5 <myopia ≤−5.0 D group and myopia >−5.0 D group. In myopia >−5.0 D group, myopia was between −5.5 D and −7.0 D. Our results indicated that there was no significant difference between pre-treatment distant and near deviation angles in myopia ≤−2.5 D group (*P* = 0.058); but in −2.5 <myopia ≤−5.0 D group (*P* = 0.001) and myopia >−5.0 D group (*P* = 0.016), there was a significant difference between pre-treatment distant and near deviation angles (Table 2).

**Table 2:** Comparison of pre-treatment distant and near deviation angle among different myopia levels (PD).

Grouping	Cases (n)	Near deviation angle	Distant deviation angle	*t* values	*P* values
Myopia ≤−2.5 D	10	45.00 ± 28.48	51.00 ± 22.33	−2.167	0.058
−2.5 <myopia ≤−5.0 D	10	21.00 ± 9.94	30.50 ± 11.16	−5.019	0.001
Myopiaå −5.0 D	5	28.00 ± 13.03	36.00 ± 12.94	−4.000	0.016

Note: PD: prism diopter.

### Comparison of pre-treatment distant and near deviation angles between type-IIAACE and type-IIIAACE

The pre-treatment distant and near deviation angles were all significantly higher in the cases with type-IIAACE than in the cases with type-IIIAACE (all *P* < 0.05, Table 3).

**Table 3:** Comparison of pre-treatment distant and near deviation angles between type-IIAACE and type-IIIAACE (PD).

	Type-IIAACE (n = 4)	Type-IIIAACE (n = 25)	*t* values	*P* values
Near deviation angle	71.25 ± 17.50	32.00 ± 22.22	3.352	0.002
Distant deviation angle	67.50 ± 20.61	39.80 ± 18.79	2.707	0.012

Notes: AACE: acute acquired comitant esotropia; PD: prism diopter.

### Comparison of distant and near deviation angles between the two groups before and after treatment

There were no significant differences in the distant and near deviation angles between the group A and group B before treatment (all *P* > 0.05). However, the distant and near deviation angles were significantly different between the group A and group B one day and one month after treatment (all *P* < 0.05); but at 6 months, the distant and near deviation angles were not significantly different between the group A and group B (all *P* > 0.05) (Table 4).

**Table 4:** Comparison of distant and near deviation angles between the group A and group B at different time points (PD).

Grouping	Pre-treatment		Post-treatment one day		Post-treatment one month		Post-treatment 6 months	
	Near	Distant	Near	Distant	Near	Distant	Near	Distant
Group A	41.92 ± 27.65	47.30 ± 22.69	14.76 ± 10.57	19.07 ± 10.24	−1.92 ± 7.13	1.69 ± 7.04	5.92 ± 7.72	6.84 ± 6.28
Group B	32.66 ± 24.11	39.66 ± 20.13	0.66 ± 3.19	3.13 ± 3.37	3.93 ± 1.83	6.20 ± 1.93	4.60 ± 2.29	5.26 ± 3.03
*t* values	0.946	0.944	4.628	5.365	−2.878	−2.237	0.595	0.866
*P* values	0.353	0.354	0.000	0.000	0.013	0.043	0.562	0.395

Note: PD: prism diopter.

### Successful rate of treatment for AACE

At post-treatment 6 months, the success rate was 84.6% (11/13) in the group A and 81.3% (13/16) in the group B, and did not display significant difference between the group A and group B (*P* = 1.00, Table 5).

**Table 5:** Comparison of various eye positions between the group A and group B at different time points [n (%)].

	Post-treatment one day	Post-treatment one month	Post-treatment 6 months
**Group A** (**n = 13**)			
Orthotropia	10 (76.2%)	10 (76.2%)	11 (84.6%)
Undercorrection	3 (23.1%)	0 (0.0%)	2 (15.4%)
Overcorrection	0 (0.0%)	3 (23.1%)	0 (0.0%)
**Group B** (**n = 16**)			
Orthotropia	16 (100%)	15 (93.4%)	13 (81.3%)
Undercorrection	0	1	3
Overcorrection	0 (0.0%)	0 (0.0%)	0 (0.0%)

Notes: Fisher exact test shows no significant difference in the orthotropia rate between the two groups at post-treatment 6 months (*P* = 1.00).

### Comparison of stereoacuity between the group A and group B at post-treatment 6 months

At post-treatment 6 months, 11 cases had near stereoacuity and 12 cases had distant stereoacuity in the group A. In the group B, all the 16 cases had both near stereoacuity and distant stereoacuity. There were no significant differences in the distant (*P* = 0.192) and near stereoacuity (*P* = 0.448) between the group A and group B at post-treatment 6 months.

### Complications after treatment

In group A, 2 patients had blephroptosis within one to 3 weeks after botulinum toxin A injection and the blephroptosis gradually returned to normal three months later without long-term limitation of eyeball movement as well as local and systemic adverse reactions. In group B, no complications occurred.

## Discussion

AACE mainly occurs in older children and adults. It is characterized by sudden-onset esotropia with diplopia, equal prism diopter in each direction and normal eye movement. The patient can accurately describe AACE onset time and diplopic condition because it occurs after well-developed binocular visual functions. Burian^1^ divided AACE into three types: type I, swan type, mostly is caused by trauma, surgery, single-eye covering during the treatment of amblyopia or single-eye vision loss; type II, Franceschtti type, is characterized by large medial oblique angle and mild hyperopia, and is usually associated with physical or psychological stress; type III, Bielschonsky type, often occurs in myopia ≤−5.00 D and uncorrected myopia. In the 29 cases of this study, the 29 all had no history of single-eye covering, 4 had emmetropia or mild hyperopia, and 25 had myopia including 20 with myopia ≤−5.00 D. Therefore, in this study, most cases had type IIIAACE, a few cases had type II AACE, and there were no the cases with type I AACE. Chen *et al*.^7^ also reported that there were no the cases with type I AACE among 47 cases with AACE.

Bielschowsky *et al*.^8^ have believed that AACE occurs in the patients with myopia ≤−5.00 D, this kind of AACE possesses distant deviation angle with mild near deviation angle or no near deviation angle. However, it has been reported that AACE also may occur in the patients with myopia >−5.00 D, and has both distant and near deviation angles^9^. Gadia *et al*.^10^ also reported that there was no significant difference between distant and near deviation angles in 15 patients with AACE. In the 25 cases with myopia of this study, 20 had myopia ≤−5.00 D and 5 myopia >−5.00 D, there was no significant difference between distant and near deviation angles in the cases with myopia ≤−2.5 D, but in the cases with myopia level >−2.5 D the distant deviation angle was significantly higher than the near deviation angle. Our results are not consistent with that reported by Bielschowsky *et al*.^8^.

Bielschowsky *et al*.^8^ also have believed that uncorrected myopia is strongly associated with type-III AACE. However, in the 25 cases with myopia, only 5 did not wear spectacles for correction of myopia. Therefore, it seems that uncorrected myopia is not related to AACE. This study also displayed that both pre-treatment distant and near deviation angles were significantly higher in the cases with type-IIAACE than in the cases with type-IIIAACE.

Botulinum toxin A has been used in the treatment of strabismus for 30 years^11^. A recent Cochrane review found that there is level I evidence supporting the efficacy of botulinum toxin for strabismus, specifically for small horizontal deviations in adults and for the retreatment of residual deviations after surgery for infantile and acquired Esotropia^12–15^. Although the direct effect of BTXA is only maintained for three months, its corrective effect on eye position can last longer^16^. The long-term corrective mechanism for eye position is unclear. Current explanations for the long-term mechanism include micro-anatomical changes in the neuromuscular junction, stretching paralyzed muscles and inhibiting the contraction of antagonistic muscles to reconstruct binocular visual function^17^, and muscle fiber remodeling^18,19^.

There are a few studies on application of botulinum toxin A in the treatment of AACE. Dawson^6^ reported that after 14 cases with AACE received botulinum toxin A injection, the deviation angle improved in 13 cases and the stereoacuity recovered in 8 cases. During the period of botulinum toxin A injection, the length-tension relationship of the antagonistic muscle was adjusted to normal state, so the eye position could be corrected^20–22^. Wu *et al*.^23^ treated different types of strabismus by botulinum toxin A injection and found that except fixed strabismus, botulinum toxin A was effective for other types of strabismus. Du *et al*.^24^ also found that botulinum toxin A injection obtained successful rate of 100% for early AACE. Wang *et al*.^25^ believed that botulinum toxin A injection may be used as an alternative treatment for AACE because 6 cases with AACE all obtained orthotropia after botulinum toxin A injection. Therefore, botulinum toxin A injection provides a new method for the treatment of AACE.

Most of previous studies are about the therapeutic effect of either botulinum toxin injection or squint correction on AACE. It is know that the procedure is simpler, the duration of anesthesia and the time in the post-anesthesia care unit are shorter, and the payment is lower in botulinum toxin injection than in squint correction. If the therapeutic effects are similar between botulinum toxin injection and squint correction, botulinum toxin injection will be a good choice for treatment of AACE. Therefore, in this study, we compared the therapeutic effects between botulinum toxin injection and squint correction on AACE.

Our results indicated that there was no statistical difference in distant and near deviation angles between the group A and group B at post-treatment 6 months, but the distant and near deviation angles were significantly higher in the group A than in group B one day and one month after treatment (Table 4). At post-treatment 6 months, the success rate was not significantly different between the group A and group B (Table 5). In the group A, undercorrection occurred in 3 cases on the post-treatment one day, with the development of the drug effect overcorrection occurred in 3 cases at post-treatment one month, and with the disappearance of the drug effect 2 cases had undercorrection again at post-treatment 6 months.

In summary, the therapeutic effect of botulinum toxin injection on AACE is similar to that of squint correction, and the procedure is simpler in botulinum toxin injection than in squint correction. Therefore, botulinum toxin injection may be a good choice for treatment of AACE. This will be further conformed by large sample. For the cases with myopia level >−2.5 D, the pre-treatment distant deviation angle is significantly higher than the pre-treatment near deviation angle. Both the pre-treatment distant and near deviation angles are greater in the cases with type-IIAACE than in the cases with type-IIIAACE. It seems that uncorrected myopia is not related to AACE.

### Subjects and methods

All study methods were approved by Institutional Review Board and Ethics Committee of the First Affiliated Hospital of Zhengzhou University and Henan Provincial Ophthalmology Hospital, and were performed in accordance with relevant guidelines and regulations. The informed consent was obtained from subjects or from a parent (for the participants under the age of 18 years).

### Subjects

AACE has been described as a dramatic onset of a relatively large angle of esotropia with diplopia and minimal refractive error.

The diagnostic criteria of AACE were (1) sudden-onset esotropia with diplopia; (2) horizontal diplopia with an equal picture interval in each direction; (3) normal eye movements in each direction; (4) a certain binocular visual function; (5) no organic lesions in the nervous system; and (6) normal corrected visual acuity.

In the Ophthalmic Center of the First Affiliated Hospital of Zhengzhou University and Henan Provincial Ophthalmology Hospital, AACE was treated by surgery before June 2016. After June 2016, botulinum toxin A injection was used to treat AACE. The inclusion criteria of this study were (1) monocular best corrected visual acuity ≥1.0; (2) no history of eye surgery; (3) esotropia ≥15 prism diopters (PD); (4) no ocular organic lesions. The exclusion criteria of this study included (1) amblyopia, nystagmus and other eye diseases; (2) other strabismus such as congenital esotropia, refractive accommodative esotropia and partial accommodative esotropia; and (3) abnormal eye movements. From January 2016 to January 2017, 40 cases with AACE were treated in the Ophthalmic Center of the First Affiliated Hospital of Zhengzhou University and Henan Provincial Ophthalmology Hospital. Of the 40 cases, 29 were in line with the inclusion and exclusion criteria. In the 29 cases, the 29 all had no history of single-eye covering, 4 had emmetropia or mild hyperopia, and 25 had myopia. Of the 25 cases with myopia, 5 did not wear spectacles for correction of myopia and 20 had a myopia ≤−5.00 D. Among the 29 cases with AACE, 13 received botulinum toxin injection (group A) and 16 squint correction (group B).

### Routine ophthalmologic examination

All cases underwent routine ophthalmologic examinations including visual acuity, outer eye, anterior segment, refracting media and eye ground to exclude ocular organic lesions. Patients wore the glasses of appropriate degree based on cycloplegic optometry, and the ocular muscle examination (the specific examination of strabismus) was carried out under myopia correction.

### Ocular muscle examination

The binocular visual function was measured using synoptophore. The cases that could identify stereoscopic pictures were regarded as having stereoacuity, otherwise as no stereoacuity. Titmus test was used to detect the near stereoacuity. The cases that passing the pattern of fly wings (3000 arc seconds) were regarded as having near stereoacuity, otherwise as no stereoacuity. The primary position deviation angle was determined by Hirschberg test. The distant (5 m) and near (33 cm) deviation angles were measured by triangular prism and alternating cover test in naked eyes and wearing spectacles. In the test-object of 5 m, the deviation angles at 25 ° upward and 25 °downward were detected to exclude A-V syndrome.

### Neurogenic examination

All patients underwent head MRI or CT to exclude nervous system disease. Head MRI or CT indicated that extraocular muscle course was normal.

### Therapeutic methods

Botulinum toxin A injection: Botulinum toxin A for injection (BTXA, 100 unit lyophilisate vials, Lanzhou Institute of Biological products, Lanzhou, China) was dissolved in 4 ml of highly purified water, after exposing the medial rectus muscle of non-fixating eye, 0.1 ml (containing 2.5 units of BTXA) was directly injected at 10 cm behind insertion point of the medial rectus muscle.

Squint correction: If the near deviation angle was more than the distant deviation angle, and the near deviation angle was between 15 and 25 PD, internal rectus recession of non-fixating eye was carried out. If the near deviation angle was less than the distant deviation angle, and the distant deviation angle was between 15 and 25 PD, lateral rectus resection of non-fixating eye was adopted. If either near deviation angle or distant deviation angle was more than 25 PD, internal rectus recession combined with lateral rectus resection of non-fixating eye was chosen. If either near deviation angle or distant deviation angle was 80 PD or more, internal rectus recession of two eyes and lateral rectus resection of non-fixating eye were performed.

### Observational items

The distant (5 m) and near (33 cm) deviation angles were measured by triangular prism and alternating cover test before treatment, as well as one day, one month and 6 months after treatment. The post-treatment total horizontal deviation of 10 PD or less was regarded as successful treatment. The primordial eye position was detected by Hirschberg test. The distant stereoacuity was determined using synoptophore and the near stereoacuity using Titmus test. The examiner of the pre-operative examination also was the observer for the outcomes of the treatment, and was masked to the treatment group.

### Statistical analysis

Statistical treatment was performed using SPSS19.0 software.Fisher exact test was used to compare successful rate between the two groups. The preoperative data were compared by one-way ANOVA and Fisher exact test. Pre-treatment and post-treatment distant and near deviation angles were compared by repeated measurement ANOVA between the two groups. The independent sample *t* test was used to compare the post-treatment stereoacuity between the two groups. Statistical significance was established at *P* < 0.05.

### Ethical approval and informed consent

All study methods were approved by the Ethics Committee. of the First Affiliated Hospital of Zhengzhou University and Henan Provincial Ophthalmology Hospital. All the subjects enrolled into the study gave written informed consent to participate.
